# Detecting early physiologic changes through cardiac implantable electronic device data among patients with COVID-19

**DOI:** 10.1016/j.cvdhj.2022.07.070

**Published:** 2022-08-04

**Authors:** Meghan Reading Turchioe, Rezwan Ahmed, Ruth Masterson Creber, Kelly Axsom, Evelyn Horn, Gabriel Sayer, Nir Uriel, Kenneth Stein, David Slotwiner

**Affiliations:** ∗ Columbia University Irving Medical Center, New York, New York; † Boston Scientific, Marlborough, Massachusetts; ‡ Weill Cornell Medicine, New York, New York

**Keywords:** Physiologic monitoring, COVID-19, Cardiac pacemaker, Implantable defibrillators

## Abstract

**Background:**

Cardiac implantable electronic devices (CIEDs) may enable early identification of COVID-19 to facilitate timelier intervention.

**Objective:**

To characterize early physiologic changes associated with the onset of acute COVID-19 infection, as well as during and after acute infection, among patients with CIEDs.

**Methods:**

CIED sensor data from March 2020 to February 2021 from 286 patients with a CIED were linked to clinical data from electronic health records. Three cohorts were created: known COVID-positive (n = 20), known COVID-negative (n = 166), and a COVID-untested control group (n = 100) included to account for testing bias. Associations between changes in CIED sensors from baseline (including HeartLogic index, a composite index predicting worsening heart failure) and COVID-19 status were evaluated using logistic regression models, Wilcoxon signed rank tests, and Mann-Whitney *U* tests.

**Results:**

Significant differences existed between the cohorts by race, ethnicity, CIED device type, and medical admissions. Several sensors changed earlier for COVID-positive vs COVID-negative patients: HeartLogic index (mean 16.4 vs 9.2 days [*P* = .08]), respiratory rate (mean 8.5 vs 3.9 days [*P* = .01], and activity (mean 8.2 vs 3.5 days [*P* = .008]). Respiratory rate during the 7 days before testing significantly predicted a positive vs negative COVID-19 test, adjusting for age, sex, race, and device type (odds ratio 2.31 [95% confidence interval 1.33–5.13]).

**Conclusion:**

Physiologic data from CIEDs could signal early signs of infection that precede clinical symptoms, which may be used to support early detection of infection to prevent decompensation in this at-risk population.

## Graphical abstract

**Figure:**
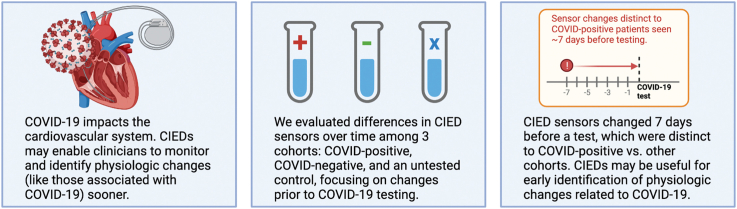

**Key Findings**

• Respiratory rate, activity, and HeartLogic index (a composite index of multiple sensors that signals potential worsening heart failure) deviated from baseline approximately 7 days before symptom onset and testing, which was significantly earlier for COVID-positive vs COVID-negative patients. Respiratory rate during the 7 days before testing significantly predicted a positive vs negative COVID-19 test, adjusting for age, sex, race, and device type, suggesting sensors may have future predictive potential.
• A higher proportion of COVID-positive patients had an implantable cardioverter-defibrillator vs other device type, indicating more severe heart failure, and on average their HeartLogic index was elevated above baseline 2 weeks prior to symptom onset and testing, which precedes the viral incubation period for COVID-19. It is possible that early sensor changes may have represented worsening heart failure status, which created a greater susceptibility to a COVID-19 infection.
• Distinct cardiac implantable electronic device sensor changes were also observed during the testing period and for several days following testing among COVID-positive compared to COVID-negative patients. Specifically, for COVID-positive patients, respiratory rate, activity, thoracic impedance, and HeartLogic index also remained significantly changed from baseline for several days or weeks after COVID-19 testing.

## Introduction

Patients with cardiovascular disease, especially heart failure, are at increased risk for contracting and suffering worse outcomes from severe acute respiratory syndrome coronavirus 2 or COVID-19.^1^^,^^2^ Early identification of infection in this high-risk cohort would be important to be able to deliver timely and more effective treatment, for example with monoclonal antibodies,^3^ and prevent further transmission. Cardiac implantable electronic devices (CIEDs) capture moment-by-moment physiologic data on cardiac patients, allowing for remote patient surveillance. Prior studies have demonstrated the power of using CIEDs to remotely monitor patients’ cardiac health at home.^4^^,^^5^ Given the well-described, broad effects of COVID-19 on the cardiac and respiratory systems,^6^ it is possible that certain CIED sensors may also detect a COVID-19 infection, potentially before symptom onset. The potential to use CIEDs to identify early signs of COVID-19 has not been well explored beyond small case series studies.^7^^,^^8^

This study seeks to characterize early physiologic changes associated with an acute COVID-19 infection among patients with CIEDs during the COVID-19 pandemic in New York City. The primary hypothesis was that significant differences in CIED sensor data would be observed during the immediate time period before COVID-19 testing for patients who tested positive compared to those who tested negative. Additionally, we aimed to characterize differences in sensor changes at the time of COVID-19 testing between patients who tested positive, tested negative, and did not receive a COVID-19 test. Finally, we aimed to compare the resolution of sensor changes after COVID-19 testing among patients who tested positive compared to those who tested negative.

## Methods

### Study design

In this cohort study, CIED sensor data were evaluated from March 2020 through February 2021 among patients at 2 large, urban, academic medical centers that are part of the NewYork-Presbyterian (NYP) Hospital network: NYP-Cornell and NYP-Columbia. Data originated from 3 sources: CIEDs, institutional COVID-19 repositories, and institutional electronic health record (EHR) data warehouses.

### Data sources

CIED data were retrieved from Boston Scientific, where data from remotely monitored permanent pacemakers, implantable cardiac defibrillators (ICDs), and cardiac resynchronization therapy (CRT) devices are stored. Boston Scientific captures and stores all sensor data from its devices, creating a longitudinal dataset for each patient over time. Ten CIED sensors were examined: respiratory rate, activity, heart rate, night heart rate, temperature, rapid shallow breathing index, thoracic impedance, heart sounds (S1, S3), and HeartLogic index.

HeartLogic is a proprietary algorithm that uses multiple sensors to track physiological trends and combines them into 1 composite index to send as a proactive alert of potential worsening heart failure.^9^ It is a cumulative index of the other sensors measured with CIEDs. The HeartLogic algorithm was previously reported to detect heart failure decompensation events with 70% sensitivity with a median of 34 days prior to an event.^9^ Because it relies on general physiologic sensors in addition to cardiac-specific sensors, HeartLogic may also predict other forms of decompensation, such as acute infection.

Some sensors (activity, respiratory rate) are measured in all patients with devices while others are only available in a subset of patients. For example, HeartLogic and its component sensors, including heart sounds and thoracic impedance, is only available for ICDs and certain types of CRT devices, and therefore only measured in patients with indications for those devices (predominantly patients with heart failure with reduced ejection fraction).

Pulse generator temperature is monitored as an internal feature of these devices at the location of the implant. It is used within the devices for component monitoring purposes, such as battery status, and is not intended to provide a measure of core body temperature. Deviations in core body temperature could be reflected in the device’s temperature measurements, but the use of this feature in this setting is investigational.

Institutional COVID-19 repositories consist of COVID-19-related data from the EHRs of all patients who have received a COVID-19 polymerase chain reaction test documented at NYP-Cornell or NYP-Columbia. COVID-19 test date and test status were obtained from institutional COVID-19 repositories.

Demographics (age, sex, race, ethnicity), clinical characteristics (body mass index, heart failure diagnosis), and events at the time of COVID-19 testing (medicine admission, intensive care unit admission, and death) were obtained from institutional EHR warehouses. We conducted a chart review of COVID-positive patients’ EHRs to determine the timing of symptom onset and types of symptoms reported as documented by clinicians in the emergency department or inpatient admission notes.

This study was approved by the Weill Cornell Medicine and Columbia University Irving Medical Center Institutional Review Boards.

### Study population

**Figure 1:**
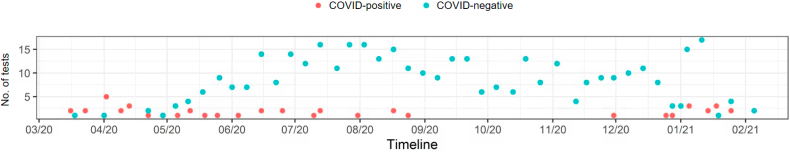
Timeline of COVID-19 test dates during the study period among patients included in the study (n = 286).

All patients who had an actively transmitting Boston Scientific CIED during the study period at NewYork-Presbyterian Hospital network, including Cornell and Columbia, and were aged 18 years or older were eligible for inclusion. Initially a total of 2210 patients meeting inclusion criteria were successfully linked to Boston Scientific and institutional EHR data. In the setting of limited available COVID-19 polymerase chain reaction tests during the first few months of the study period, institutional testing protocols were designed to prioritize testing for patients with a probable COVID-19 infection based on presenting signs and symptoms. Therefore, during part of the study period, COVID-negative patients were acutely ill enough to warrant a test. The timeline of when the COVID-19 tests were administered to patients in the study by test result (positive vs negative) is shown in Figure 1. The greater proportion of COVID-positive tests from March through May of 2020, and subsequent shift towards a greater proportion of COVID-negative tests thereafter, likely reflects these changes in institutional testing protocols as tests became more widely available and asymptomatic patients were able to receive tests.

To account for this testing bias in the first few months of the study period, we created a cohort of 100 randomly selected untested “true” control patients. Thus, 3 cohorts of patients were created using COVID-19 test date and status documented in the institutional COVID-19 repositories: (1) patients with a positive COVID-19 test (“COVID-positive”), (2) patients with a temporally concordant negative COVID-19 test (“COVID-negative”), and (3) patients who had a Boston Scientific CIED but did not have a COVID-19 test (“control”).

### Statistical analysis

Characteristics of each cohort were described using frequencies and percentages or means and standard deviations for categorical and continuous variables, respectively. Ten CIED sensors were examined in each analysis. Baseline parameters were computed using average sensor data from 60 to 30 days before COVID-19 testing for each patient. Pretest parameters were computed as the average sensor data during the 7 days preceding COVID-19 testing for all sensors except temperature, for which we selected the maximum value instead of the average. Event parameters were computed using maximum temperature and average of other sensors during the 15-day window surrounding COVID-19 testing.

The number of days before COVID-19 testing that the sensors considerably differed from baseline was identified for each sensor. We determined *a priori* that a sensor value is considered significantly different from baseline if it increased or decreased 1 standard error above or below that patient’s baseline and continued to remain different until the COVID-19 test date. This ensures that all days following the first significant day are also significantly different. The number of days after COVID-19 testing until sensors returned to baseline values was identified for each sensor was also identified using 1 standard error difference.

The primary comparison was between COVID-positive and COVID-negative patients. This comparison allowed changes that were specific to COVID-19 infection, and not other causes of acute decompensation, to be identified. As a sensitivity analysis to account for testing bias, COVID-positive patients were also compared to 100 randomly selected untested control patients. In the absence of a COVID-19 test to anchor the analytic window for control patients, a 15-day window between March 1 and July 1, 2020, was randomly selected to serve as a simulated event. This time frame was selected because it includes the first COVID-19 shutdown period in New York City, allowing possible changes in activity, breathing, heart rate, and other sensors that may be due to either stay-at-home orders, change in activity levels, or overall stress and anxiety owing to the pandemic.

The number of days before COVID-19 testing that the sensors significantly differed from baseline and the number of days that elapsed after a test before sensors returned to baseline were compared between COVID-positive and COVID-negative patients. Logistic regression models were used to identify whether the presence of sensor changes greater than 1 standard deviation in the 7 days before a test were associated with test status (positive vs negative). Multivariable model selection included screening univariate sensor associations (*P* < .5) and adjusting for age, sex, race, and device type. Wilcoxon signed rank tests were used to compare sensor changes from baseline to event within cohorts (paired test). Mann-Whitney *U* tests were used to compare the magnitude of mean change in each sensor between cohorts.

All statistical analyses were conducted using R statistical software (R Core Team, Vienna, Austria). A data engineer from Boston Scientific (R.A.) conducted all statistical analyses because of his knowledge and prior experience analyzing and interpreting the volume and variety of data collected from CIED sensors. The data engineer met weekly with 2 researchers not employed by the company (M.R.T., D.S.) to discuss the ongoing study, identify appropriate statistical analyses, and interpret findings. All raw data and analytic files were shared with the 2 researchers for transparency purposes. Furthermore, all coauthors met regularly to discuss the ongoing analysis and contribute insights from their clinical expertise and experience caring for cardiac patients with COVID-19. Therefore, we made a concerted, conscientious effort to ensure research findings were interpreted in the context of clinical relevance only.

## Results

### Patient characteristics

**Figure 2:**
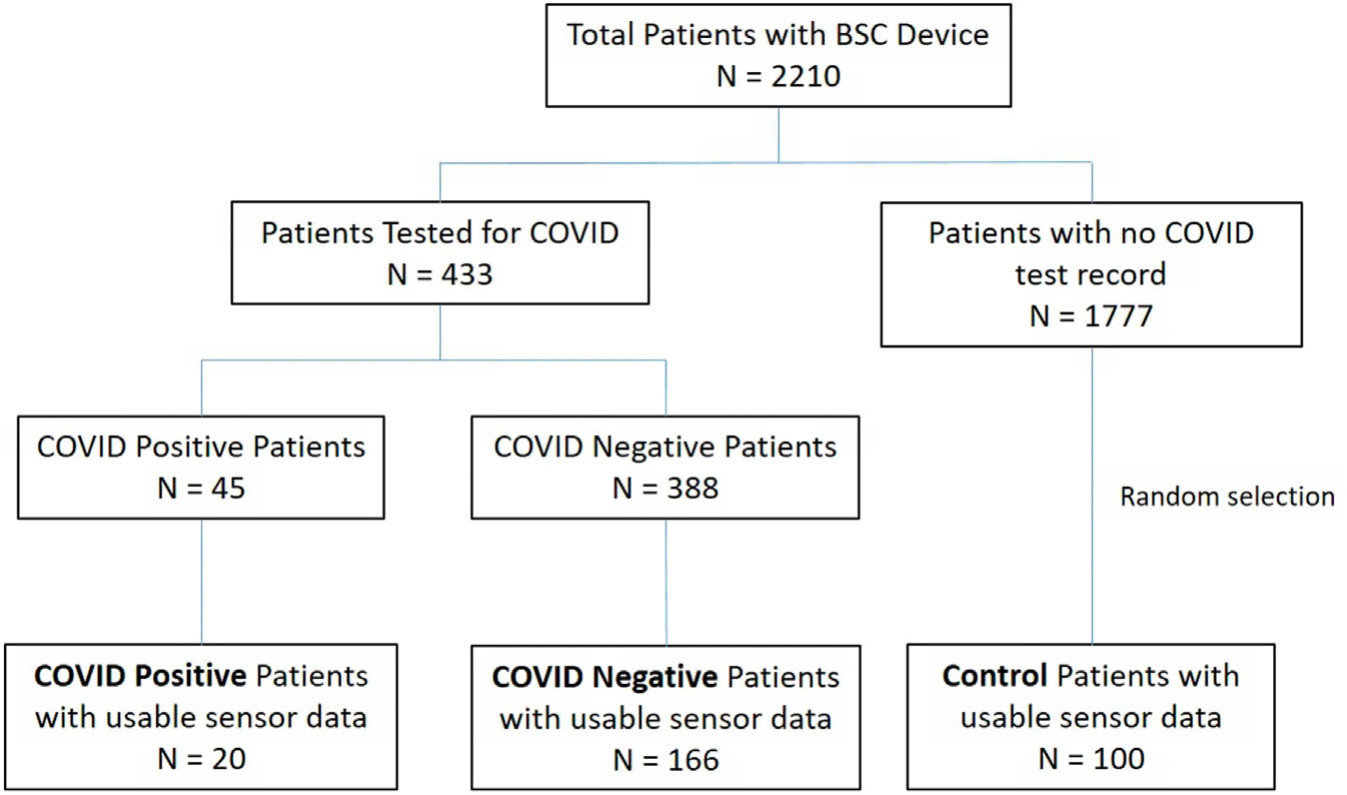
Sampling strategy of patient cohorts: COVID-positive (n = 20), COVID-negative (n = 166), and untested controls (n = 100).

Of the 2210 patients meeting eligibility criteria, 433 had a COVID-19 test result in the institutional repositories during the study period and 1777 did not (Figure 2). Of the patients with COVID-19 tests, 45 tested positive and 388 tested negative. Twenty COVID-positive and 166 COVID-negative patients had usable CIED sensor data; these formed the cohorts used in this analysis. The remaining patients were excluded because they did not have usable sensor data, either because the device data were not available in the remote server owing to missed transmissions or because the timeframe of data transmission did not overlap with the test date (for example, devices implanted after a COVID-19 test). Additionally, the 100 randomly selected control patients were selected from the pool of 1777 patients without a COVID-19 test in the institutional repositories.

Demographic characteristics of the 3 cohorts are reported in Table 1. Comparing COVID-positive vs COVID-negative vs control patients, significant differences existed by race (White: 25% vs 52% vs 42%; Black/African American: 20% vs 18% vs 19%; Other: 35% vs 14% vs 11% [*P* = .04]) and ethnicity (Hispanic/Latino: 45% vs 19% vs 11% [*P* = .004]). Age, sex, and body mass index did not significantly differ between the 3 cohorts.

**Table 1:** Demographic characteristics of COVID-positive, COVID-negative, and control patient cohorts

	COVID-positive (n = 20)	COVID-negative (n = 166)	Control (n = 100)	*P* value†
Age	68.2 (13.3)	71.3 (15.1)	67.9 (14.8)	.18
Sex				.31
Male	10 (50)	98 (59)	61 (61)	
Female	10 (50)	68 (41)	37 (37)	
Other/unknown	0 (0)	0 (0)	2 (2)	
Race				.04∗
White	5 (25)	86 (52)	42 (42)	
Black / African American	4 (20)	30 (18)	19 (19)	
Asian	1 (5)	14 (8)	10 (10)	
Other	7 (35)	23 (14)	11 (11)	
Unknown	3 (15)	13 (8)	18 (18)	
Ethnicity				.004∗
Hispanic/Latino	9 (45)	32 (19)	11 (11)	
Non-Hispanic/Latino	9 (45)	107 (65)	64 (64)	
Unknown	2 (10)	27 (16)	25 (25)	
BMI	30.3 (7.0)	27.8 (5.4)	27.0 (5.1)	.09
Unknown	3 (15)	48 (29)	43 (43)	
Heart failure	7 (35)	74 (45)	36 (36)	.48
Unknown	0	10	7	
Device type				<.001∗
CRT	3 (15)	35 (21)	28 (28)	
ICD	13 (65)	48 (29)	47 (47)	
Pacemaker	4 (20)	83 (50)	25 (25)	
Medicine admission‡	16 (80)	53 (32)	---	<.001∗
Unknown	0 (0)	12 (7)	---	
ICU admission‡	0 (0)	5 (3)	---	.86
Unknown	3 (18)	22 (13)	---	
Death‡	0 (0)	0 (0)	---	.37
Unknown	0 (0)	12 (7)	---	

Values are mean (SD) or n (%) test for categorical measures.

Statistically significant *P* values are indicated by an asterisk.

BMI = body mass index; CRT = cardiac resynchronization therapy; ICD = implantable cardioverter-defibrillator; ICU = intensive care unit.

† *P* values were calculated using ANOVA for continuous measures and χ^2^

‡ Refers to events at the time of the COVID-19 test. Medicine admissions exclude surgical admissions for which routine preoperative COVID-19 testing was conducted. Not evaluated for the control group, who did not have a COVID-19 test.

There were also significant differences by device type. Comparing COVID-positive vs COVID-negative vs control, more COVID-positive patients had an ICD (65% vs 29% vs 47%) and fewer COVID-positive patients had a pacemaker (20% vs 50% vs 25%). There was no difference in the proportion of patients diagnosed with heart failure between the cohorts. A higher proportion of COVID-positive patients had a medical admission at the time of COVID-19 testing compared to COVID-negative (80% vs 32%, *P* < .001), but there was no difference in intensive care unit admissions or deaths.

Heart failure status by device type is reported in Supplemental Table 1. The majority of patients with CRT devices (83%) or ICDs (67%) were diagnosed with heart failure, whereas few patients with pacemakers were (22%).

COVID-19 symptom information for COVID-positive patients is reported in Supplemental Table 2. The most commonly reported symptoms were cough (65%), dyspnea (55%), and subjective fever/chills (35%). The median symptom onset before COVID-19 testing was 3 (IQR 3–7) days prior to COVID-19 testing.

### Early sensor changes prior to COVID-19 testing

**Figure 3:**
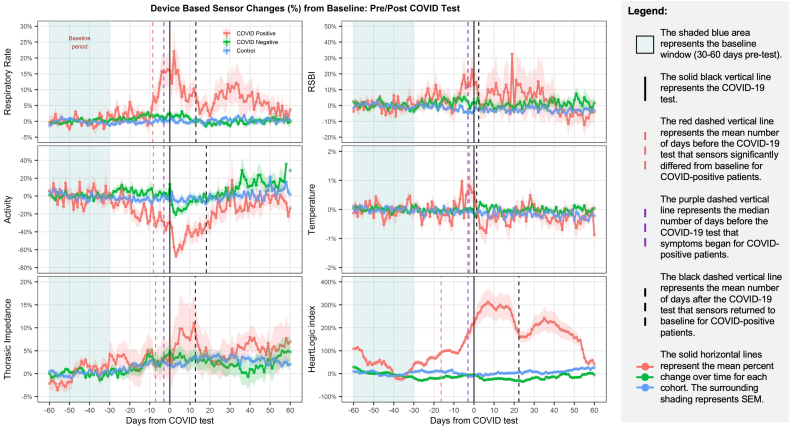
Average sensor changes among COVID-positive (n = 20), COVID-negative (n = 166), and control (n = 100) patient cohorts.

Figure 3 shows the average sensor changes among the 3 patient cohorts from 60 days prior through 60 days following COVID-19 testing. The figure illustrates sensor changes distinct to COVID-positive patients; specifically, for COVID-positive patients, several sensors (respiratory rate, activity, thoracic impedance, and HeartLogic index) significantly changed from baseline several days prior to symptom onset, and all sensors significantly changed from baseline prior to COVID-19 testing. Respiratory rate, activity, thoracic impedance, and HeartLogic index also remained significantly changed from baseline for several days or weeks after COVID-19 testing.

The illustrated unique sensor changes associated with COVID-positive patients and not COVID-negative patients were confirmed in statistical tests. Table 2 compares the number of days before COVID-19 testing that the sensors changed more than 1 standard deviation from baseline for COVID-positive and COVID-negative patients. Multiple sensors changed sooner for COVID-positive compared to COVID-negative patients: HeartLogic index (mean 16.4 vs 9.2 days; *P* = .08), respiratory rate (mean 8.5 vs 3.9 days; *P* = .01), activity (mean 8.2 vs 3.5 days; *P* = .008), and temperature (mean 2.3 vs 1.0 days; *P* = .08).

**Table 2:** Average number of days prior to COVID-19 testing with significant changes from baseline, comparing COVID-positive to COVID-negative patients

Sensors	COVID-positive (n = 20)		COVID-negative (n = 166)		*P* value
	N†	Number of days (mean ± SD)	N†	Number of days (mean ± SD)	
Respiratory rate	14	-8.5 ± 8.5	113	-3.9 ± 7.5	.01∗
Activity	18	-8.2 ± 8.3	154	-3.5 ± 6.1	.008∗
Night heart rate	8	-2.1 ± 2.8	35	-2.3 ± 4.2	.80
24-Hour heart rate	15	-3.5 ± 4.3	98	-3.3 ± 5.1	.62
Temperature‡	17	-2.3 ± 2.7	139	-1.0 ± 1.5	.08
RSBI	8	-3.1 ± 4.0	35	-4.3 ± 8.9	.75
Thoracic impedance	8	-7.1 ± 11.1	35	-11.4 ± 11.3	.29
Heart sound S1	8	-2.6 ± 4.6	34	-3.0 ± 5.5	.80
Heart sound S3	8	-2.6 ± 3.5	33	-1.3 ± 2.6	.29
HeartLogic index	8	-16.4 ± 12.5	33	-9.2 ± 12.6	.08

Mann-Whitney *U* test (Wilcoxon rank sum test) was used to compare COVID-positive to COVID-negative.

Statistically significant *P* values are indicated by an asterisk.

RSBI = rapid shallow breathing index.

† N represents the number of patients with devices containing specified sensors and with valid sensor value during baseline and test period.

‡ Temperature represents the pulse generator temperature at the location of the implant.

In unadjusted multivariable logistic regression models, respiratory rate, activity, and temperature significantly predicted a positive COVID-19 test (Table 3). After adjusting for age, sex, race, and device type, respiratory rate during the 7 days before COVID-19 testing significantly predicted a positive COVID-19 test (odds ratio: 2.31 [95% confidence interval 1.33–5.13]).

**Table 3:** Odds ratios and 95% confidence intervals for mean cardiac implantable electronic device sensor changes during the 7 days prior to COVID-19 testing associated with COVID-19 test status (positive vs negative patients), unadjusted and adjusted for age, sex, race, and device type

Sensor	Unadjusted OR (95% CI)	Adjusted OR (95% CI)
Respiratory rate	1.75 (1.29–2.44)∗	2.31 (1.33–5.13)∗
Activity	0.36 (0.15–0.85)∗	2.88 (0.32–27.4)
Night heart rate	1.06 (0.92–1.21)	---
24-Hour heart rate	1.01 (0.90–1.14)	---
Temperature†	2.15 (1.40–3.49)∗	2.96 (0.97–10.99)
RSBI	1.36 (0.99–1.96)	---
Thoracic impedance	0.99 (0.85–1.19)	---
Heart sound S1	0.53 (0.05–2.98)	---
Heart sound S3	212.90 (0.12–833264.27)	---
HeartLogic index	1.09 (1.00–1.22)	---

Odds ratios indicated by asterisks are statistically significant. Only variables with significant associations with the outcome variable in unadjusted models were included in adjusted models.

CI = confidence interval; OR = odds ratio; RSBI = rapid shallow breathing index.

† Temperature represents the pulse generator temperature at the location of the implant.

### Sensor changes at the time of COVID-19 testing

Among COVID-positive patients, we observed statistically significant changes in respiratory rate (15% increase), temperature (1% increase), HeartLogic index (227% increase), and activity (44% decrease) from baseline to event (Supplemental Table 3). Among COVID-negative patients, we observed statistically significant changes in activity (12% decrease) from baseline to event. There was a significantly greater mean change for respiratory rate, activity, temperature, and HeartLogic index among COVID-positive patients compared to COVID-negative patients. In the sensitivity analysis, we observed statistically significant changes in activity (6% decrease) and temperature (0.3% increase) in the control patient cohort from baseline to event (Supplemental Table 4). There was a significantly greater mean change for respiratory rate, activity, temperature, and HeartLogic index among COVID-positive patients compared to control patients.

### Sensor changes following COVID-19 testing

Table 4 compares the number of days after COVID-19 testing before sensors returned to baseline between COVID-positive and COVID-negative patients.

**Table 4:** Average number of days after COVID-19 testing that sensors returned to baseline, comparing COVID-positive to COVID-negative patients

Sensors	COVID-positive (n = 20)		COVID-negative (n = 166)		*P* value
	N†	Number of days (mean ± SD)	N†	Number of days (mean ± SD)	
Respiratory rate	14	13.0 ± 16.5	112	4.0 ± 9.6	.006∗
Activity	18	18.2 ± 20.5	154	6.1 ± 12.4	.007∗
Night heart rate	8	4.5 ± 8.3	35	4.5 ± 12.0	.67
24-Hour heart rate	15	6.7 ± 10.4	98	5.1 ± 11.5	.23
Temperature‡	17	1.3 ± 1.3	139	1.5 ± 2.6	.35
RSBI	8	2.4 ± 3.1	35	3.7 ± 10.9	.67
Thoracic impedance	8	12.8 ± 20.5	35	11.9 ± 16.5	.75
Heart sound S1	8	13.1 ± 21.1	34	4.6 ± 13.4	.79
Heart sound S3	8	3.1 ± 3.2	33	2.4 ± 6.5	.23
HeartLogic index	8	22.4 ± 21.5	33	13.5 ± 22.0	.08

Mann-Whitney *U* test (Wilcoxon rank sum test) was used to compare COVID-positive to COVID-negative.

RSBI = rapid shallow breathing index.

† N represents the number of patients with devices containing specified sensors and with valid sensor value during baseline and test period.

‡ Temperature represents the pulse generator temperature at the location of the implant.

Multiple sensors returned to baseline later for COVID-positive compared to COVID-negative patients: HeartLogic index (mean 22.4 vs 13.5 days; *P* = .08), activity (mean 18.2 vs 6.1 days; *P* = .007), and respiratory rate (mean 13.0 vs 4.0 days; *P* = .006).

## Discussion

This is the first study to systematically evaluate the early physiological changes prior to the onset of an acute COVID-19 infection indirectly detected by CIEDs. We found that respiratory rate, activity, and HeartLogic index deviated from baseline approximately 7 days before symptom onset and testing, which was unique to COVID-positive patients, and respiratory rate during the week before testing predicted a positive test. Distinct CIED sensor changes were also observed during the testing period and for several days following testing among COVID-positive compared to COVID-negative patients.

It is possible that the early changes in CIED sensors may have represented worsening heart failure status, and that patients may have been more likely to later develop moderately or severely symptomatic COVID-19 in the setting of worsening heart failure. HeartLogic index, which was originally developed to predict heart failure decompensation, was elevated above baseline 2 weeks prior to symptom onset and testing among COVID-positive patients–which precedes the viral incubation period for COVID-19.^10^ Several prior case reports have described sharp increases in HeartLogic index in patients with a confirmed COVID-19 infection.^11^, ^12^, ^13^ This possibility is also supported by the higher proportion of COVID-positive patients with an 10.13039/100007866ICD vs other device type, suggesting severe heart failure requiring an 10.13039/100007866ICD may be associated with a greater susceptibility to a COVID-19 infection. While the HeartLogic algorithm was created to provide early warning of possible heart failure exacerbation, by integrating data from multiple physiologic sensors and monitoring for variations, the algorithm may be triggered by other physiological changes. Therefore, it is important for clinicians to be cognizant of the fact that an elevated HeartLogic index may indicate other acute illnesses, such as sepsis.

We present evidence that the observed sensor changes are in some way associated with COVID-19 infection because the magnitude of change was significantly different for COVID-positive vs COVID-negative and control patients. For instance, although activity significantly decreased among all patient cohorts, likely owing to stay-at-home orders limiting activity for all New York City residents as observed in prior studies,^14^ we observed a significantly greater decrease in activity for COVID-positive patients. Similarly, we observed distinctly different sensor changes between COVID-positive and COVID-negative patients. Because testing protocols in the early phase of the study period meant that only acutely ill patients received a test during these first months, COVID-negative patients may have been acutely ill with another condition, such as a heart failure exacerbation. In fact, we observed elevated HeartLogic index in both groups. Nonetheless, the sensor changes including HeartLogic index were more dramatic for COVID-positive patients, suggesting unique physiologic changes for these patients that were detected by CIEDs.

Therefore, our findings provide early evidence of the power of remote monitoring with CIEDs for the early detection of physiologic changes that may be subtle and may precede clinical symptoms. Prior case studies have demonstrated CIED sensor changes at the time of a COVID-19 infection^7^ and noted an increase in device-detected respiratory rate at symptom onset, prior to a COVID-19 diagnosis.^12^^,^^15^ Building on these early observations, this cohort study with 286 patients provided robust evidence that these changes occurred before symptom onset and diagnosis, and were detected by CIEDs. In particular, others have noted, and our study supports, the potential to use HeartLogic index to actively monitor and identify perhaps both heart failure–related and non–heart failure–related decompensation early in the course of disease.^11^, ^12^, ^13^ This index measures the magnitude of change in an array of sensors, most notably activity and respiratory rate, that are captured by a broad range of CIEDs and even commercially available devices, suggesting a number of devices may be useful in monitoring cardiovascular disease patients during the pandemic. While some of the other CIED vendors measure transthoracic impedance to monitor for heart failure exacerbations, only Boston Scientific displays and integrates data from multiple physiologic sensors, integrates the data into an algorithm, and has approval from the U.S. Food and Drug Administration to alert clinicians. The early detection capabilities of machine learning algorithms leveraging data from CIEDs (such as HeartLogic index) may also be extended to identify other types of non–heart failure–related decompensation, such as influenza or pneumonia infections, among high-risk groups. Thus, while CIEDs have traditionally been used for cardiac monitoring, including during the pandemic to reduce exposure associated with avoidable in-person cardiac care,^4^^,^^5^^,^^16^^,^^17^ this study suggests CIEDs may also be used for surveillance and early detection of other forms of acute decompensation, including but not limited to COVID-19. The ability to follow a high-risk cohort of cardiac patients remotely and receive alerts when they may be in the early stages of an infection may significantly improve clinicians’ early intervention abilities in clinical care.

We also observed unique footprints of a more prolonged post-COVID recovery through CIED sensors; respiratory rate and HeartLogic index remained higher and activity remained lower for weeks following the test date for COVID-positive patients. Already numerous studies have reported a range of cardiac-specific postacute sequelae of COVID-19 infection related to persistent cardiac injury, placing patients at elevated risk of heart failure, myocardial infarction, myocarditis, pericarditis, and arrhythmia.^6^^,^^18^ CIED data offer continuous longitudinal measures of physiologic function following COVID-19 infection and thus may be a powerful tool in characterizing true cardiac injury or exacerbation of underlying cardiopulmonary status resulting from the infection, especially when paired with other clinical datasets, including those originating from EHRs.

Despite a small number of COVID-positive patients, the overall size of the sample including the COVID-negative and control cohorts is a strength of this study, relative to previous case series examining CIED data among COVID-positive patients.^11^^,^^13^ Given that New York City was an early epicenter of COVID-19 infections in the United States,^19^ we were able to accrue and follow a cohort of COVID-19 patients with CIEDs for a longer period of time compared to regions of the United States that experienced their first surges of COVID-19 later.

Limitations include limited generalizability of the sample, the small number of positive patients, and the small number of sensors that could be measured in the entire sample owing to sensor differences between device types. It is possible that the sensors that were available in all devices were also significantly different between cohorts (eg, respiratory rate and activity). It is possible that we may have detected significant differences in other sensors if we had been able to examine more devices that measured a wider range of sensors. Specifically, there were no significant differences on a number of sensors between COVID-positive and COVID-negative patients that we may have been able to detect with greater statistical power. Future work determining differences in sensors changes between larger cohorts of COVID-positive and COVID-negative patients, including those with devices that measure a range of sensors, are needed. Larger cohorts could also be used to build and validate a predictive classifier of COVID status using sensor data, which we were unable to do because of the small number of positive cases. An additional limitation is classification bias owing to rapid changes in institutional testing protocols and accuracy of COVID-19 tests during the study period, together with a lack of access to COVID-19 tests performed outside our institutions. Therefore, patients in the COVID-negative or control cohorts may have actually been infected with COVID-19. Finally, the data analyst was unable to remain blinded to the study question or COVID test status because of the data science needed to create the 3 cohorts, which may have introduced bias into the analysis.

In conclusion, physiologic data from remotely monitored CIEDs provide a unique opportunity to detect physiologic changes that may be associated with COVID-19 infection early in the course of illness. Sensor changes in COVID-positive patients signal early signs of infection that precede clinical symptoms, which may be used to support early detection of infection to prevent decompensation in this at-risk population.

## Disclaimer

Given his role as Section Editor, David Slotwiner had no involvement in the peer review of this article and has no access to information regarding its peer review.

## Funding Sources

This project was funded through a research grant from 10.13039/100008497Boston Scientific Corporation. M.R.T. is funded through NIH/10.13039/100000056NINR (K99NR019124).
