# Supplementary material for: Detecting early physiologic changes through cardiac implantable electronic device data among patients with COVID-19
Source: Cardiovasc Digit Health J. 2022 Aug 4;3(5):247–55. doi: 10.1016/j.cvdhj.2022.07.070 (PMC9349024; doi:10.1016/j.cvdhj.2022.07.070)
Supplement: Supplemental material [file mmc1.docx]

**SUPPLEMENTAL MATERIAL**

| **Supplemental Table 1.** Heart failure status by device type, n(%) | | | | |
| --- | --- | --- | --- | --- |
|  | CRT (n=66) | ICD (n=108) | Pacemaker (n=112) | P-value |
| HF | 55 (83%) | 72 (67%) | 25 (22%) | <0.001 |
| No HF | 6 (9%) | 30 (28%) | 81 (72%) |  |
| Unknown | 5 (8%) | 6 (5%) | 6 (5%) |  |
| *CRT: Cardiac resynchronization therapy; ICD: Implantable cardioverter defibrillator, HF: Heart Failure.* | | | | |

| **Supplemental Table 2.** Symptom onset and prevalence among COVID-positive patients (n=20) | |
| --- | --- |
| Symptom onset (median [interquartile range] days)* | 3.0 (3.0 - 7.0) |
| Symptom prevalence (n[%]) |  |
| Cough | 13 (65%) |
| Dyspnea | 11 (55%) |
| Subjective fever/ chills | 7 (35%) |
| Weakness | 4 (20%) |
| Diarrhea | 4 (20%) |
| Aches | 4 (20%) |
| Nausea | 3 (15%) |
| Fatigue | 3 (15%) |
| Chest pain | 1 (5%) |
| Loss of speech or movement | 1 (5%) |
| Rhinorrhea | 1 (5%) |
| Sore throat | 1 (5%) |
| Headache | 1 (5%) |
| Loss of taste or smell | 1 (5%) |
| **Missing for 7 patients.* | |

| **Supplemental Table 3.** Comparison of cardiac implantable electronic devices (CIED) sensor data between COVID-positive and COVID-negative during the 15-day window surrounding the COVID-19 test | | | | | | | | | | | | | | |
| --- | --- | --- | --- | --- | --- | --- | --- | --- | --- | --- | --- | --- | --- | --- |
| **Sensors** | **COVID-positive (n=20)** | | | | | | **COVID-negative (n=166)** | | | | | | **Positive vs. negative** | |
|  | N | Baseline  Mean (Median) | Event  Mean  (Median) | Changes  (Mean ± SEM) | Pct. change | P-value | N | Baseline  Mean (Median) | Event  Mean  (Median) | Changes  (Mean ± SEM) | Pct. change | P-value | P-value |  |
| Respiratory Rate | 15 | 16.16 (16.14) | 18.65 (17.36) | 2.49 ± 0.61 | 15% | **0.0026*** | 116 | 17.40 (17.24) | 17.66 (17.57) | 0.26 ± 0.14 | 2% | 0.10 | **0.0003** |  |
| Activity | 19 | 1.44 (1.03) | 0.80 (0.69) | -0.64 ± 0.18 | -44% | **0.0001*** | 160 | 1.47 (1.20) | 1.29 (1.06) | -0.18 ± 0.04 | -12% | **<0.0001*** | **0.0009** |  |
| Night Heart Rate | 8 | 76.06 (80.21) | 78.05 (78.20) | 1.99 ± 2.63 | 3% | 0.67 | 38 | 72.59 (73.84) | 72.81 (73.58) | 0.21 ± 0.75 | 0.3% | 0.76 | 0.85 |  |
| 24-hr Heart Rate | 15 | 73.01 (68.72) | 75.60 (76.58) | 2.59 ± 1.48 | 4% | 0.10 | 101 | 74.70 (75.00) | 75.61 (74.63) | 0.91 ± 0.47 | 1% | **0.03*** | 0.24 |  |
| Temperature | 18 | 97.39 (97.62) | 98.34 (98.99) | 0.95 ± 0.31 | 1% | **0.0095*** | 144 | 96.32 (95.87) | 96.21 (95.95) | -0.11 ± 0.09 | 0.1% | 0.09 | **0.0011*** |  |
| RSBI | 8 | 15.01 (14.19) | 16.46 (15.83) | 1.45 ± 0.92 | 10% | 0.09 | 38 | 13.88 (13.40) | 13.97 (13.48) | 0.08 ± 0.34 | 0.6% | 0.99 | 0.27 |  |
| Thoracic Impedance | 8 | 48.62 (49.85) | 50.44 (51.81) | 1.81 ± 2.17 | 4% | 0.33 | 38 | 44.13 (46.43) | 45.41 (46.53) | 1.28 ± 0.70 | 3% | **0.02*** | 0.66 |  |
| Heart Sound S1 | 8 | 2.29 (2.35) | 2.34 (2.18) | 0.05 ± 0.24 | 2% | 0.67 | 37 | 2.96 (2.78) | 2.97 (2.71) | 0.02 ± 0.09 | 0.6% | 0.89 | 0.47 |  |
| Heart Sound S3 | 8 | 0.95 (0.93) | 1.03 (0.97) | 0.08 ± 0.06 | 9% | 0.21 | 36 | 1.00 (0.89) | 0.95 (0.86) | -0.04 ± 0.02 | -4% | 0.06 | 0.06 |  |
| HeartLogic Index | 8 | 4.49 (1.92) | 14.71 (11.48) | 10.21 ± 3.98 | 227% | **0.01*** | 36 | 7.39 (5.31) | 6.59 (3.83) | -0.80 ± 1.50 | -11% | 0.70 | **0.01*** |  |
| *A Wilcoxon test was used for all comparisons. RSBI: Rapid shallow breathing index.* | | | | | | | | | | | | | | |

| **Supplemental Table 4.** Comparison of cardiac implantable electronic devices (CIED) sensor data between COVID-positive and control patients during the 15-day window surrounding the COVID-19 test | | | | | | | | | | | | | | | |
| --- | --- | --- | --- | --- | --- | --- | --- | --- | --- | --- | --- | --- | --- | --- | --- |
| **Sensors** | **COVID-positive (n=20)** | | | | | | **Control patients (n=100)** | | | | | | | **Positive vs. Control** | |
|  | N | Baseline  Mean (Median) | Event  Mean  (Median) | Changes  (Mean ± SEM) | Pct. change | P-value | N | Baseline  Mean (Median) | Event  Mean  (Median) | Changes  (Mean ± SEM) | Pct. change | P-value | P-value | |  |
| Respiratory Rate | 15 | 16.16 (16.14) | 18.65 (17.36) | 2.49 ± 0.61 | 15% | **0.0026*** | 81 | 16.85 (16.47) | 16.95 (16.75) | 0.10 ± 0.10 | 0.6% | 0.36 | **0.002*** | |  |
| Activity | 19 | 1.44 (1.03) | 0.80 (0.69) | -0.64 ± 0.18 | -44% | **0.0001*** | 99 | 1.64 (1.50) | 1.54 (1.28) | -0.10 ± 0.04 | -6% | **0.02*** | **0.01*** | |  |
| Night Heart Rate | 8 | 76.06 (80.21) | 78.05 (78.20) | 1.99 ± 2.63 | 3% | 0.67 | 50 | 70.62 (69.31) | 70.11 (67.33) | -0.51 ± 0.51 | -0.7% | 0.33 | 0.38 | |  |
| 24-hr Heart Rate | 15 | 73.01 (68.72) | 75.60 (76.58) | 2.59 ± 1.48 | 4% | 0.10 | 73 | 74.62 (73.59) | 73.94 (71.86) | -0.68 ± 0.42 | -0.9% | 0.11 | 0.05 | |  |
| Temperature | 18 | 97.39 (97.62) | 98.34 (98.99) | 0.95 ± 0.31 | 1% | **0.0095*** | 95 | 97.41 (97.61) | 97.07 (97.09) | -0.33 ± 0.06 | -0.3% | **0.0001*** | **0.0007*** | |  |
| RSBI | 8 | 15.01 (14.19) | 16.46 (15.83) | 1.45 ± 0.92 | 10% | 0.09 | 50 | 13.43 (13.94) | 13.41 (14.08) | -0.02 ± 0.16 | -0.1% | 0.91 | 0.16 | |  |
| Thoracic Impedance | 8 | 48.62 (49.85) | 50.44 (51.81) | 1.81 ± 2.17 | 4% | 0.33 | 50 | 46.38 (46.84) | 46.68 (46.34) | 0.30 ± 0.31 | 1% | 0.34 | 0.51 | |  |
| Heart Sound S1 | 8 | 2.29 (2.35) | 2.34 (2.18) | 0.05 ± 0.24 | 2% | 0.67 | 50 | 3.09 (2.93) | 3.05 (2.82) | -0.04 ± 0.03 | -1.2% | 0.20 | 0.72 | |  |
| Heart Sound S3 | 8 | 0.95 (0.93) | 1.03 (0.97) | 0.08 ± 0.06 | 9% | 0.21 | 50 | 0.85 (0.82) | 0.85 (0.83) | 0.00 ± 0.01 | 0.4% | 0.72 | 0.23 | |  |
| HeartLogic Index | 8 | 4.49 (1.92) | 14.71 (11.48) | 10.21 ± 3.98 | 227% | **0.01*** | 50 | 4.04 (1.39) | 4.13 (2.49) | 0.09 ± 1.05 | 2% | 0.93 | **0.04*** | |  |
| *A Wilcoxon test was used for all comparisons. Control patients did not have a COVID-19 test; therefore a randomly selected 15-day window within the study period was used.* *RSBI: Rapid shallow breathing index.* | | | | | | | | | | | | | | | |
